# Management of a Previously Failed Root Perforation Repair with Geristore and Deepithelialized Gingival Graft: A 5-Year Follow-Up Case Report

**DOI:** 10.1155/2023/7335196

**Published:** 2023-09-27

**Authors:** Talal M. Zahid

**Affiliations:** Department of Periodontology, King Abdulaziz University, Jeddah, Saudi Arabia

## Abstract

Iatrogenic root perforation presents a significant management challenge for clinicians as it may seriously harm the periodontium. More specifically, perforations occurring relative to the crestal bone have a poor prognosis even after repair due to their proximity to the gingival tissues. The current literature reports the use of various materials for root perforation repair including calcium hydroxide, glass ionomer cement, amalgam, and mineral trioxide aggregate (MTA), to name a few. This case report describes the clinical management of a cervical perforation that occurred on the maxillary central incisor. The perforated area was initially repaired with MTA but failed after one year, which resulted in an active lesion at the midlabial aspect of the tooth. The case was subsequently treated using a resin-modified glass ionomer cement (Geristore®) and deepithelialized free gingival graft (DGG). There were favorable clinical and radiographic outcomes at 1-, 3-, and 5-year follow-up. The use of DGG, however, led to some late complications such as gingival cul-de-sac and color discrepancy, which were later resolved with gingivoplasty and frenectomy. We thus conclude that Geristore® has the potential to be a better repair material than the existing ones for crestal and subcrestal root perforations.

## 1. Introduction

Root perforation refers to an opening or a hole that communicate between the root canal system and periodontal space. Both pathologic and iatrogenic communications can cause root perforations. Pathological perforations often result from a pathologic process such as severe dental caries or root resorptive defects [1]. Routine clinical examination usually reveals these perforations. In contrast, iatrogenic perforations often occur due to procedural errors.

These can arise during various stages of endodontic treatment, especially during postspace preparation, root canal instrumentation, or access cavity formation [2].

In clinical practice, root perforations most commonly result from iatrogenic causes, accounting for about 2 to 12% of all failed endodontic cases [3–5]. According to a recent systematic review, however, the occurrence of iatrogenic root perforations ranges from 0.6% to 17.6% [6]. A primary reason for perforations during endodontic treatment is the lack of proper knowledge, experience, and care in the preparation of access cavity and postspace. Other risk factors for perforation include tooth morphology, tooth type, and tooth location [2, 6]. Kvinnsland et al. estimated that about 53% of iatrogenic perforations occur during postplacement and the remaining 47% occur during endodontic instrumentation [7]. The authors also found that the maxillary teeth are more commonly affected by perforations than the mandible. Contrarily, Tsesis et al. reported a higher prevalence of perforations in mandibular molars compared to other dental locations [8].

Iatrogenic root perforations may cause serious damage to the periodontium. This is due to the high likelihood of bacterial infection at the perforation site, which may halt the healing process. Once this infectious process begins, it may trigger various inflammatory responses. These responses may lead to the loss of periodontal tissue and alveolar bone, the formation of granulomatous tissue, the proliferation of epithelial cells, and, finally, the appearance of a periodontal pocket [1, 2]. Delay in diagnosing and treating root perforations can lead to further complications and possibly tooth loss. Therefore, for the optimal management of root perforation, early diagnosis and prompt treatment are crucial [9].

Given the severity of root perforations, the ideal repair material should excel in sealing, osteogenesis, and cementogenesis, while also being biocompatible, bacteria-resistant, and cost-effective [2, 10]. Although no single material meets all these criteria, several bioceramics, including mineral trioxide aggregate (MTA), biodentine, EndoSequence, BioAggregate, and calcium-enriched mixture (CEM), have ushered in a transformative era in endodontics [10–13]. MTA, the pioneer in this field since its introduction in 1993, offers excellent sealing and biocompatibility due to its hydrophilic particles like tricalcium silicate [14, 15]. Biodentine builds on this foundation with enhanced adaptability, particularly in challenging root conditions [12]. EndoSequence adds stability and biocompatibility with its blend of calcium silicate and calcium phosphate, while BioAggregate offers resilience under varying pH conditions [12, 13, 16]. CEM is notable for its calcium-rich formula, which may enhance hydroxyapatite formation near the exposed periodontium [13]. Amidst these repair materials, Geristore® emerges as a noteworthy addition. It is a biocompatible, self-adhesive, resin-modified glass ionomer with less moisture sensitivity than traditional glass ionomer cement. Geristore® serves multiple roles, from acting as a root-end filler to repairing subgingival anomalies such as root surface caries and iatrogenic perforations [17–21].

In this case report, we discussed managing a failed MTA repair of an iatrogenic root perforation. We corrected the repair, partially replacing MTA with Geristore® to leverage its sealant properties and soft tissue graft adhesion capabilities. Additional aspects include managing gingival cul-de-sac and color discrepancies. The perforation occurred on the midlabial aspect of a maxillary central incisor, just apical to the cementoenamel junction. The case was followed-up for approximately 5 years. Clinical and radiographic data showed the successful outcome of the surgery performed.

## 2. Case Report

**Figure 1:**
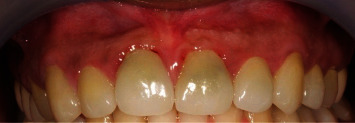
Clinical view of upper anterior crowns before root canal retreatment.

A 30-year-old Saudi female patient had undergone root canal retreatment and temporary crown placement on the maxillary right central incisor (#11; FDI tooth notation system) by a restorative dentist in 2015. She originally came to replace her preexisting upper anterior crowns due to greyish discoloration and cosmetic concerns (Figure 1). The restorative dentist removed the existing old crowns and replaced them with temporary crowns. Retreatment for root canal at tooth #11 was done successfully.

**Figure 2:**
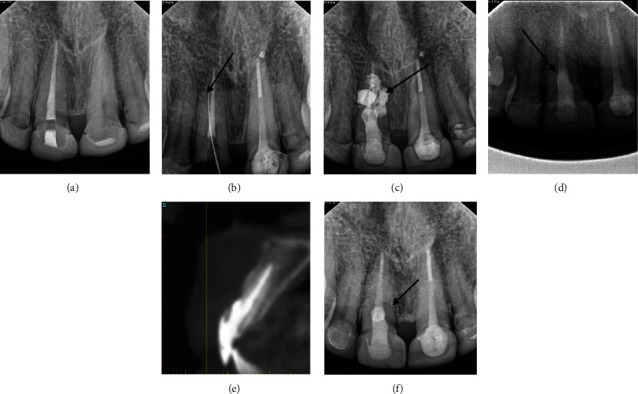
(a) Radiograph after root canal retreatment. (b) Radiograph showing root perforation after postspace preparation. (c) Calcium hydroxide placement. (d) MTA placement. (e) Cone beam CT scan 18 months after MTA (prior to our intervention). (f) Follow-up after 5 years of Geristore® placement.

The treatment plan consisted of internal bleaching, followed by postspace preparation and finally the new crown fabrication for tooth #11. A perforation, however, occurred at the midlabial aspect of a maxillary central incisor during postspace preparation. The restorative dentist then placed calcium hydroxide as a temporary measure and referred her to an endodontist for further evaluation and treatment. The endodontist planned mineral trioxide aggregate (MTA) repair to seal the perforation and prevent microleakage. The choice of MTA also supports tissue healing due to its biocompatible nature, providing additional benefit in the repair process. A coronally positioned flap was designed. The flap was raised, exposing 3 × 3 mm of perforation, and calcium hydroxide was removed. The endodontist then placed MTA and repositioned the flap. One month later, the final crown was placed and cemented.  Figure 2 presents radiographs from the initial root perforation repair and post-MTA placement, a CT scan performed 18 months after MTA, and a follow-up radiograph 5 years post-Geristore® placement.

**Figure 3:**
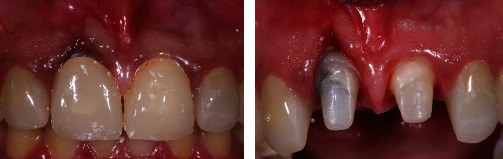
The patient presents with a bleeding, deep pocket, pus, and recession at tooth #11.

About 18 months later, the patient was referred to the Department of Periodontology clinic in the King Abdulaziz University Dental Hospital for the management of gingival recession. She presented with a chief complaint of gingival bleeding, sensitivity, and discomfort around tooth #11. Upon periodontal examination, gingival recession, deep pocketing, and pus discharge were observed (Figure 3). A black line was also observed at the crown-gingival junction. This might have resulted from several factors including root discoloration from previous treatment and perforation, gray discoloration from porcelain-fused-to-metal (PFM) restorations, and a thin gingival biotype causing recession.

At the time of clinical presentation, she was in good physical condition without any history of systemic disease. Clinical examination and intraoral radiographs revealed a recession of 3 mm (Miller classification I) with a 7 mm probing depth at the midlabial aspect of tooth #11. There were no signs of periapical pathology and coronal leakage in relation to the tooth; hence, retreatment of the tooth was not recommended. Based on the persistent discomfort and infection, we suspected a failure of the previous endodontic root perforation repair. While further confirmation through 3D imaging or exploratory surgery would have been ideal, the lack of a CT scan in the previous treatment may have been due to availability or the operator's decision. However, in our case, a CT scan was deemed essential to thoroughly evaluate treatment options, including the potential need for a dental implant. Based on clinical signs, we opted to address the gingival recession with a deepithelialized free gingival graft (DGG) and repair the perforation using Geristore®.

## 3. Surgical Repair with DGG and Geristore®

**Figure 4:**
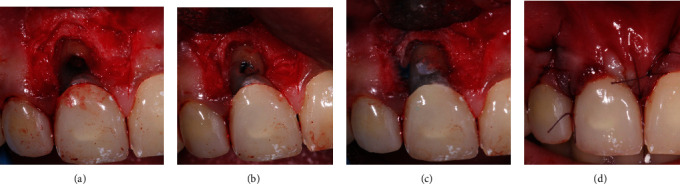
(a) Exposure of root surface and bone after flap reflection. (b) Removal of MTA. (c) Geristore® cement was placed over the MTA. (d) Suturing with 4.0 Vicryl sutures.

The patient was provided with a comprehensive explanation of the diagnosis, potential outcomes, and available treatment alternatives, and she expressed her preference to retain the tooth. Informed consent was obtained prior to the periodontal surgery. The procedure involved local anesthesia (2% lidocaine with 1 : 100,000 epinephrine), and a coronally positioned flap was designed using two vertical incisions. The flap was partially thickness at the papilla to provide a bed for the soft tissue graft and full-thickness mucoperiosteal in the remaining area to expose the root surface and bone. Additional flap mobility was achieved with a periosteal releasing incision, and the frenum tissue was internally incised for optimal coverage. The area was evaluated with methylene blue to detect any fractures or crack lines, which were not found. The superficial layer of the MTA was partially removed, and Geristore® cement was applied over the MTA following the manufacturer's guidelines. Excess luting cement from previous procedures on the tooth's buccal surface was removed, and the surface was smoothed using a flame-shaped finishing bur to contour the Geristore® cement, ensuring a smooth root surface.  Figure 4 illustrates the key steps involved in the surgical repair of tooth #11.

The surgical site was then irrigated with a sterile saline solution. The DGG was harvested from the palate following the technique described by Zucchelli et al. [22] A 15 surgical blade was used to completely remove the epithelial tissue and deepithelialize the graft. The flap was positioned coronally at the level of the cementoenamel junction (CEJ) to ensure maximum coverage of the DGG. Both the root surface and the Geristore® cement were fully covered by the DGG during placement. The flap was sutured using a 4.0 resorbable suture called Vicryl (Polyglactin 910). No periodontal dressing was provided. It was deemed unnecessary as the sutures provided adequate wound stability and protection, making postoperative cleaning easier. She was advised to avoid brushing the treated site for 14 days. The patient was prescribed an antimicrobial rinse (0.2% chlorhexidine gluconate) twice daily for 2 weeks and a pain medication (ibuprofen 400 mg) for 3 days.

**Figure 5:**
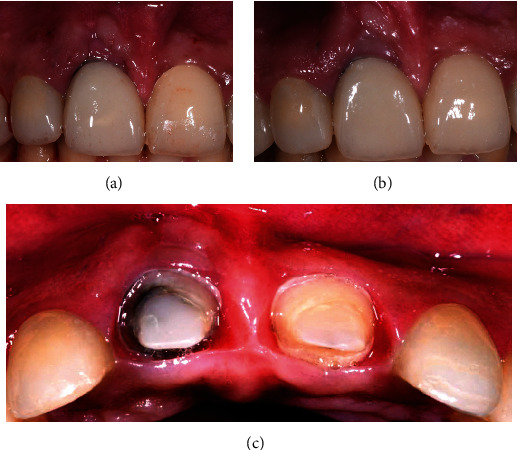
(a) 2-week follow-up. (b, c) 6-week follow-up; identification of gingival cul-de-sac.

After the surgical intervention, the patient underwent periodic follow-ups biweekly over a period of six weeks. During this time, the patient was totally asymptomatic and did not report any unfavorable events at all visits. At the 2-week follow-up visit, sutures were removed. Intraoral examination revealed satisfactory periodontal healing. At the 4-week follow-up visit, the flap appeared to be retracted, and the DGG was exposed. Despite this observation, the subsequent 6-week follow-up showed evidence of healing and tissue remodeling. The DGG also seemed stable. However, a gingival cul-de-sac was noted (Figure 5).

## 4. Management of Gingival Cul-De-Sac and Color Discrepancy

**Figure 6:**
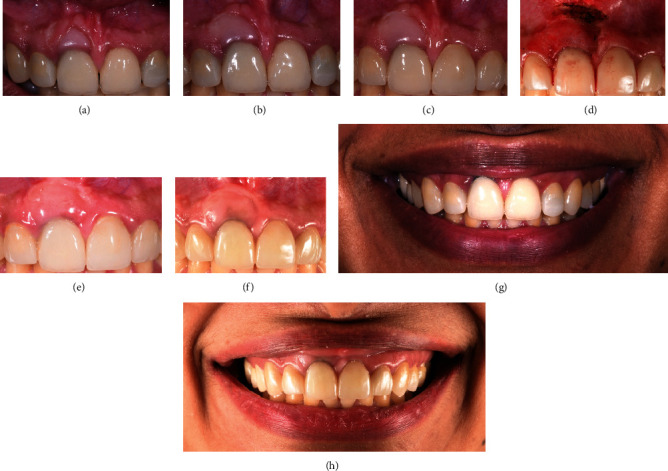
(a) One-year follow-up after surgical placement of Geristore®. (b) Four-month follow-up after gingivoplasty. (c) 18-month follow-up after the gingivoplasty. (d) Laser frenectomy and gingivoplasty were performed. (e) 6-week follow-up after frenectomy. (f) One-year follow-up after frenectomy. (g) Maximum smile before starting the treatment. (h) Maximum smile line after the treatment.

One year after the initial healing, the patient returned to the clinic with the complaint of a color mismatch. Clinical examination revealed no signs of bleeding or periodontal pathology. The exposed DGG appeared thicker, suggesting maturation and remodeling of the integrated graft. The flap appeared more retracted than the previous year. A clear demarcation of the gingival cul-de-sac was also noted (Figure 6(a)). Gingivoplasty was carried out using a large diamond bur to blend the demarcated border and treat the cul-de-sac.

About 18 months after the gingivoplasty, the patient returned to our clinic with concerns about the mesial demarcation (Figure 6(c)). Clinical examination revealed good blending of the lateral demarcation but no improvement at the mesial area. Stippling of the gingiva was apparent at the gingival margin. A creeping attachment was also noted at the tooth, which covered the crown.

About 10 months later, a second frenectomy was performed. During the initial perforation repair, only the labial frenum muscular tissue was released internally. The outer mucosal tissue was kept intact to aid flap closure and graft coverage. The second frenectomy involved complete tissue excision to create an even area and promote more uniform healing. In addition to the frenectomy, gingivoplasty for the whole area was done using laser and diamond round bur (Figure 6(d)). Follow-up at 6 weeks revealed no demarcation with the naked eye (Figure 6(e)). At one-year follow-up, the surgical site was fully healed with no visible demarcation (Figure 6(f)).

The black line at the crown-gingival junction was treated using an all-ceramic restoration with a high opacity coping (Figure 6(g)). We also discussed internal bleaching and subgingival margin preparation with the patient. None of them, however, were chosen in the end. The perforation made internal bleaching unfeasible, and we avoided subgingival margin preparation to protect the biological width and the perforation border. The all-ceramic restoration was chosen after the patient's consent. It resulted in a more confident smile and faint black shadow in the postoperative image (Figure 6(h)). This approach successfully balanced biological, functional, and esthetic considerations, eliminating the need for extraction or implant. The patient was satisfied with her smile, and the color mismatch was much better. Periapical radiographs also showed no underlying issues.

## 5. Discussion

Iatrogenic root perforation poses significant clinical challenges for the prognosis of endodontic treatment. It is likely to instigate the formation of granulation tissue, by triggering inflammatory responses in the periodontium, which may cause the loss of attachment or, sometimes, the tooth itself [1, 2]. Three of the most important variables affecting the prognosis include (1) the level of perforation, (2) the timing of intervention, and (3) the sealing ability of the selected repair material [9, 11].

Perforations occurring relative to the crestal bone and the epithelial attachment often have the worst prognosis. Fuss and Trope named this region as the critical zone [9]. The furcation region of multirooted teeth is also considered within this zone because of its relative closeness to the junctional epithelium and gingival sulcus [23]. When perforation occurs in the critical zone, its proximity to the gingival tissues can lead to contamination with oral cavity bacteria. This bacterial influx into the perforation can result in gingival downgrowth of epithelium and ultimately rapid pocket formation. Once the alveolar bone is severely damaged, granulation tissue may develop and thereby invaginate into the tooth via the perforation tract [1, 2, 11].

On the other hand, perforations in the apical and middle thirds of the root have a better prognosis than those in the cervical third or the furcation regions [24]. The likely reasons for this are that these regions are more easily accessible, carry less risk of bacterial entry into the perforated area, and do not involve the periodontium while sealing with repair materials [2, 9].

In general, a better prognosis is usually associated with timely repair of the perforation than with delayed care. In fact, the defect needs to be sealed immediately to avoid an undue influx of bacteria from the oral cavity. Delaying the sealing may worsen the prognosis and even result in tooth loss [1, 11]. Taken together, considering everything discussed above, it can be said that cases with new, small, apical, and middle perforations are most likely to have a favorable prognosis than those occurred within the critical zone.

In the present case, CT scan radiographs revealed a subcrestal perforation on the midlabial aspect of a maxillary central incisor during postspace preparation. It posed a management challenge as cervical perforations usually have a poor prognosis after repair. We initially chose MTA for perforation repair, given its well-established efficacy in promoting reparative dentin formation. Although MTA is a good sealant and has been the material of choice for root perforation repairs [16], there have been multiple reports of its failures in the literature [25–27]. In our case, the patient returned with an active lesion 18 months after the perforation repair, indicating poor sealing with MTA. This failure might be due to poor adhesion of the soft tissue to the material. The remnant luting excess might also play role in the suboptimal sealing by MTA. Subsequently, we employed Geristore®, a resin-modified glass ionomer, over the MTA to act as a sealer and facilitate soft tissue graft adhesion. This combination strategy was aimed at maximizing the benefits of both materials, with MTA promoting reparative dentin formation and Geristore® offering a reliable seal and a surface for soft tissue adhesion.

Geristore® has remarkable histological biocompatibility. Dotto et al. [21] suggested it as the material of choice for crestal and subcrestal perforations. Unlike MTA, the Geristore® cement does not have a mud-like consistency and is not prone to dissolution. It also has comparatively higher compressive strength than MTA, which is needed for the repair of furcal region or cervical third perforations [10, 17, 18, 25]. Geristore® cement was also chosen for the current clinical case because of its physical characteristics and fluoride release capacity. In addition, the fact that it is light-cured facilitates its use. Geristore® forms strong chemical bonds with the calcium ions in dentin, which may be the reason why it adheres to dentin so well [19, 21]. On the other hand, the high fluidity of its cement encourages better flow, which results in better perforation cavity filling and improved sealing [28]. To sum up, these characteristics collectively imply that Geristore® is a suitable material for root perforation repairs, especially in the cervical and furcal regions where the need for such a restorative material is very high [21].

In the current case, DGG was used instead of subepithelial connective tissue graft (SCTG) for the management of gingival recession. DGG involves obtaining a free (epithelialized) gingival graft (FGG) and then deepithelizing it outside the mouth in order to be used as a CTG [22]. We chose it because it is a more recent technique, easier to obtain and perform, and less likely to shrink postoperatively than SCTG. DGG is also generally appreciated for its better color match compared to FGG [29]. However, in our case, color variation was observed. Such color discrepancy could be attributed to patient-specific factors or postoperative changes, such as individual healing responses and tissue characteristics. Alternatively, a microscopic remnant of the previous epithelium not completely removed could also be a contributing factor.

On the other hand, the possible complications associated with DGG is less explored in the current literature. A recent randomized clinical trial by Ripoll et al. [30], however, noted a number of late complications with the use of DGG, including graft reepithelialization, change in graft color, cul-de-sac, epithelial bands, superficial revascularization, epithelial cysts, and bone exostoses. Some of these complications happened in our case as well, which were later resolved with gingivoplasty and laser frenectomy. Ripoll et al. [30] also reported that such complications occurred only with the use of DGG but not with CTG. They concluded that, given the occurrences of late complications, DGG seemed to be a less secure method of treating gingival recession than CTG. Our case provides further evidence in support of their conclusion.

To the best of our knowledge, this was the first case of its kind where a patient with a subcrestal root perforation was followed up for almost 5 years, from initial root canal treatment to addressing gingival cul-de-sac and color discrepancy. For a good prognosis, a thorough assessment of radiographs, knowledge of dental morphology, proper training in handling repair materials, and a long-term follow-up are the key elements in the treatment of iatrogenic perforation. By following this guidance, practitioners can significantly lessen the need for subsequent interventions that might result in an uncertain prognosis.

## 6. Conclusion

Subcrestal root perforations can result in a poor prognosis of the affected tooth even after perforation repair due to persistent periradicular inflammation. Geristore®, owing to its high sealing ability and superior physical characteristics, appears to be a better repair material than the existing ones for the management of cervical root perforations. However, in the present case, the use of DGG for root coverage was associated with several late complications including graft reepithelialization, gingival color mismatch, cul-de-sac, and epithelial bands; hence, utmost caution should be exercised while preparing the DGG to ensure full epithelial removal. Finally, as case reports are often considered the lowest level of evidence, more methodologically sound studies need to be conducted to establish the findings of this case report.

## Data Availability

Data are available on request.
